# Dislocation Engineered PtPdMo Alloy With Enhanced Antioxidant Activity for Intestinal Injury

**DOI:** 10.3389/fchem.2019.00784

**Published:** 2019-11-15

**Authors:** Wei Long, Xiaoyu Mu, Jun-Ying Wang, Fujuan Xu, Jiang Yang, Jingya Wang, Si Sun, Jing Chen, Yuan-Ming Sun, Hao Wang, Xiao-Dong Zhang

**Affiliations:** ^1^Tianjin Key Laboratory of Radiation Medicine and Molecular Nuclear Medicine, Institute of Radiation Medicine, Chinese Academy of Medical Sciences and Peking Union Medical College, Tianjin, China; ^2^Department of Physics and Tianjin Key Laboratory of Low Dimensional Materials Physics and Preparing Technology, School of Science, Tianjin University, Tianjin, China; ^3^State Key Laboratory of Oncology in South China Collaborative Innovation Center for Cancer Medicine Sun Yat-sen University Cancer Center, Guangzhou, China

**Keywords:** PtPdMo, radiation protection, intestinal injury, antioxidant, radiotherapy

## Abstract

Radiotherapy is the mainstay for abdomen and pelvis cancers treatment. However, high energy ray would inflict gastrointestinal (GI) system and adversely disrupt the treatment. The anti-oxidative agents provide a potential route for protecting body from radiation-induced injuries. Herein, highly catalytic nanocubes with dislocation structure are developed for treatment of intestinal injury. Structural and catalytic properties show that Mo incorporation can enhance antioxidant activity by dislocation structure in the alloy. *In vitro* studies showed that PtPdMo improved cell survival by scavenging radiation-induced ROS accumulation. Furthermore, after animals were exposed to lethal dose of radiation, the survival was increased by 50% with the PtPdMo i.p. treatment. Radioprotection mechanism revealed that PtPdMo alleviated the oxidative stress in multi-organs especially the small intestine by inhibiting intestinal epithelium apoptosis, reducing DNA strands breaks and enhancing repairing ability. In addition, PtPdMo protected hematopoietic system by improving the number of bone marrow and peripheral blood cells.

## Introduction

Radiotherapy is employed in treating many advanced gynecological cancers (Yoshida et al., 2016; Mabuchi et al., 2017). Ionizing radiation is delivered around the target site to destroy and prevent recurrence of malignancy (Song et al., 2017). As long-term survival improves, pelvis radiation is associated with increased risk of gastrointestinal (GI) tract damages which can lead to severe GI symptoms, and radiation-induced chronic intestinal disorder can extend for years after therapy (Kuku et al., 2013; Shadad et al., 2013). Additionally, ionizing radiation also inflicts hematopoietic system causing lethal bone marrow depletion, triggering p-53 dependent multi-organ deficits (Gudkov and Komarova, 2003; Rafii et al., 2016; Wen et al., 2016; Racioppi et al., 2017). For protecting normal intestine tissues from radiation injuries in radiotherapy, constructive strategies were tested in cell or animal experiments for the future clinical use (Hua et al., 2012, 2017; Rotolo et al., 2012; Vendetti et al., 2017; Wei et al., 2018).

Radiation can cause direct cell death of the rapidly proliferating crypt epithelium, which triggers excessive inflammatory responses (Gubernatorova et al., 2017). High energy radiation causes water radiolysis and forms free radicals that could constantly attack macromolecules in cells, resulting in protein carbonylation, lipid peroxide, and DNA strand breaks (Robbins and Zhao, 2004; Spitz et al., 2004; Azzam et al., 2012; Datta et al., 2012). Accumulation of ROS is beyond the internal repair ability and will consequently result in hematopoietic suppression and intestinal epithelium loss, which are susceptible to inflammations and infections. Unfortunately, catalysts with high ROS scavenging ability are still in short supply. As a consequence, the need to alleviate radiation induced side effects during therapy is still unmet.

Metal catalysts with prolonged blood circulation showed high efficiency in decomposing ROS *in vivo* (Colon et al., 2009, 2010; Walkey et al., 2015). In our previous works, various metal catalysts protected animals from radiation by scavenging excessive ROS, which could increase the survival rate after subjected to lethal radiative dose (Zhang et al., 2016b, 2017; Wang J. et al., 2018). Meanwhile, we also reported that the antioxidant property of catalysts could be improved by proper modification to achieve better radioprotective effects with enhanced ROS scavenging ability (Wang J. Y. et al., 2018). Inspired by that, catalytic ternary alloy nanocubes engineered with surface defects are prepared to prevent radiation-induced damages.

**Figure 1:**
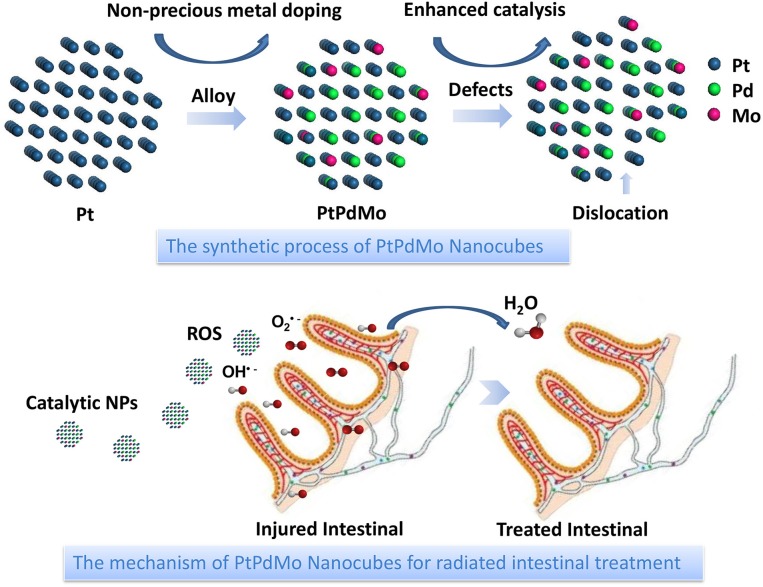
Schematic illustration of PtPdMo protected intestinal epithelium against radiation damages.

Herein, the polyvinylpyrrolidone (PVP) coated PtPdMo nanocubes is presented as an antioxidant to improve the survival rate by alleviating radiation-induced intestinal and hematopoietic damages (Figure 1). PVP was used as a reducing agent thanks to its weak reducing power and a steric stabilizer to protect the PtPdMo nanocubes from agglomeration. Results show that the survival fraction of mice exposed to 7.2 Gy, 662 keV gamma-ray increased to 50% after PtPdMo i.p. treatment. As a contrast, mice without PtPdMo treatment failed to survive (0% survival rate). Further studies reveal that PtPdMo ameliorated the oxidative stress both *in vitro* and *in vivo* by reducing radiation-induced ROS accumulation. Moreover, PtPdMo treatment can inhibit intestinal epithelium loss and hematopoietic deficits, as well as liver injuries.

## Materials and Methods

### PtPdMo Synthesis

All the constitutes were purchased from Sigma-Aldrich. According to the previous study (Wang J. Y. et al., 2018), the syntheses of Pt, PtPd, PtPdMo nanocubes were followed by the same procedure except for the constitution of ingredients: Pt(acac)_2_ (60.0 mg); Pt(acac)_2_ (30.0 mg), Pd(acac)_2_ (25.0 mg); Pt(acac)_2_ (20.0 mg), Pd(acac)_2_ (16.0 mg), MoO_2_(acac)_2_ (16 mg) with PVP (160.0 mg), and DMF (10 mL) were mixed together in NaI solution (2 mL, 0.15 g mL^−1^) in an autoclave, heated to 150°C for 10 h. The reaction system was then cooled down to room temperature. The precipitate was washed by acetone and ethanol, and centrifuged at 12,000 rpm for 10 min to remove excess reactants. Then the precipitate was suspended with PBS (pH 7.4) and centrifuged 3 times for the following characterization and biology experiments. In the simple one-pot synthesis process, polyvinylpyrrolidone (PVP) was used as a reducing agent thanks to its weak reducing power and a steric stabilizer to protect the PtPdMo nanocubes from agglomeration. Moreover, PVP-coated PtPdMo nanocubes possess better stability, water solubility and biocompatibility, which is beneficial to minimize their side effects *in vivo* and *in vitro*.

### Materials Characterization

TEM and HRTEM were conducted on JEM-2100F (JEOL, Japan) operated at 200 kV. The valence states of elements were characterized by PHI XPS (Perkin Elmer, USA) with the Al Kα excitation. Hydrodynamic diameter of PtPdMo was measured by Malvern Zetasizer nano ZS90 after being dissolved in 10% FBS solution for 24 h.

### Peroxidase-Like Activity Evaluation

All the agents were provided by the Assay Kit (Rapid ABTS method; S0121, Beyotime). ABTS and H_2_O_2_ mixture was incubated with 1 mg/mL Pt, PtPd, and PtPdMo solution at room temperature for 1 h, protected from the light. Then the solution was centrifuged at 12,000 rpm for 10 min to remove nanocubes, and the absorption value was detected by a UV–vis spectrophotometer (UV3600, Shimadzu, Japan) at 414 nm. The PtPdMo at different concentrations was measured at 405 nm by a microplate spectrophotometer (CMax Plus, Molecular Devices, China).

### *In vitro* Radiation Protection

Chinese hamster ovary cells (CHO) cells were purchased from National Infrastructure of cell line resource, cultured in DMEM with 10% FBS, and supplied with 5% CO_2_ in humidity. CHO cells were seeded into 96 or 6-well plates and kept in an incubator overnight. Cell toxicity was assessed using 96-well plates with 410^3^ cells per well, and cells were incubated for 24 h. PtPdMo nanocubes (0~300 μg/mL) were added into each well and kept incubating for another 24 or 48 h. Then cell viability was tested by MTT assay and calculated by the ratio of OD value in treated groups to control group. For radiation protection studies, cells reaching 70~80% adherent rate in wells were incubated with PtPdMo nanocubes for 50 min and subjected to a series of radiation ranging from 2 to 6 Gy. 24 h later, survival at the dose of 4Gy was assessed using MTT assay. Intracellular ROS was detected by DCFH-DA fluorescent probe as discussed before (Wang J. et al., 2018). Colony forming under different radiation doses was performed 7 days later. The colonies were fixed in methanol for 5 min, followed by staining with Giemsa solution at room temperature for 30 min. Colonies that consisted of more than 50 cells were countered. Data were fitted to the single- hit, multitarget model using Origin software version 9.0.

### *In vivo* Radiation Protection

All animals were purchased and handled according to guidelines approved by the Animal Studies Committee of Institute of Radiation Medicine, Chinese Academy of Medical Sciences (CAMS). C57 male mice at 6–8 weeks were divided into radiation, rad+Pt, rad+PtPd, and rad+PtPdMo group (*n* = 10). The mice were intraperitoneally injected with 50 mg/kg Pt, PtPd, and PtPdMo nanocubes 30 min before 7.2 Gy whole body radiation (^137^Cs with activity of 3,600 Ci and photon energy of 662 KeV). Survival rates of all groups were recorded during 30 days.

### *In vivo* Radiation Protection Mechanism

We assigned 36 mice to the following groups: control for 1 day, radiation for 1 day and rad+PtPdMo for 1 day; control for 7 day, radiation for 7 day and rad+PtPdMo for 7 day (*n* = 6). After exposed to 6 Gy radiation, mice were sacrificed at 1 and 7 days. Blood samples were used for hematology and biochemistry studies. Intestines and livers were collected and homogenized in PBS. Tissue homogenate was employed for super oxide dismutase (SOD) and Malondialdehyde (MDA) test according to the manufacture's instruction (Beyotime). Besides, bone marrow cells from bilateral femora was flushed to PBS or 0.005 M CaCl_2_ solution. Bone marrow nucleated cells suspended in PBS were tested by a blood cell counter (Mindray, BC-2800Vet). Other bone marrow samples were placed under 4°C for 30 min and centrifuged at 2,500 rpm for 15 min. The precipitation was suspended in 0.2 M perchloric acid and kept in boiling water for 15 min. After filtering, DNA absorption was detected at 268 nm by a UV-vis spectrophotometer.

### Tunnel Assay and Immunochemistry

Intestines were fixed in 10% formalin for 48 h, then made into paraffin sections. The slides were immersed in dimethylbenzene and gradient ethanol, then blocked with proteinase K at 37°C for 15 min. Cy3-labeled terminal deoxynucleotidyl transferase (TdT) was dropped onto the slides, kept in 37°C for 60 min. Finally, the sections were counterstained with DAPI and mounted with anti-quench agent. Intestine paraffin sections were processed according to a routine H&E procedure. Meanwhile, other slides were added with 3% H_2_O_2_ to inactivate endogenous peroxidase, then proceeded to heat-mediated antigen retrieval (pH 6.0). After tissues were blocked with 10% BSA for 40 min, primary anti-γ-H2AX and PCNA antibodies were incubated with tissue at 4°C overnight. The slides were rinsed in TBST for 3 times and incubated with biotinylated secondary antibody for 1 h. Antigens were detected by DAB and washed with water. Slides were immersed in gradient ethanol and dimethylbenzene reversely, then mounted with neutral balsam. The percentage of broken villi and positive staining area were analyzed by ImageJ software.

### Statistical Analyses

All data presented in this study were the mean ± SD of experiments repeated three times or more, and were analyzed by one-way ANOVA or Student's *t*-test.

## Results and Discussion

### Dislocation Structure Was Formed by Pd and Mo Doping

**Figure 2:**
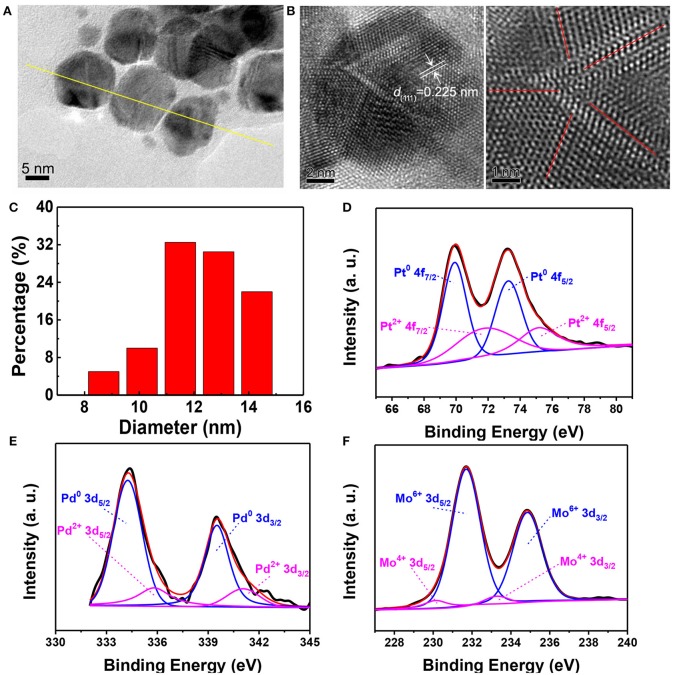
**(A)** TEM image of PtPdMo nanocubes with an average size of 12.08 nm. **(B)** HRTEM image of PtPdMo nanocubes. The interplanar spacing of 0.225 nm is corresponding to (111) crystal plane, and the dislocation exists along the bounded ridge of nanocrystal labeled by red lines. **(C)** Hydrodynamic test of PtPdMo dissolved in 10% FBS at the concentration of 1 mg/mL. XPS spectra of **(D)** Pt 4f, **(E)** Pd 3d, and **(F)** Mo 3d in the PtPdMo nanocubes, respectively.

The catalytic PtPdMo nanocubes was engineered in the base of Pt nanocubes to form the dislocation structure by doping with Pd and Mo elements. The as-synthesized PtPdMo nanocubes was employed to protect intestine from radiation-induced oxidative stress (Figure 1). TEM image displayed in Figure 2A suggests that the average size of PtPdMo is 11.5 ± 0.5 nm. The lattice spacing within an individual nanocubes is 0.225 nm corresponding to (111) plane, which is slightly less than the face-centered-cubic (FCC) PtPd alloy crystal (~0.23 nm) due to the Mo doping (Huang et al., 2016; Zheng et al., 2018). The dislocation structure of PtPdMo is presented in Figure 2B marked by red lines, and the enlarged exposing area by surface defects can increase the catalytic activity than its Pt and PtPd counterparts. Hydrodynamic diameter of PtPdMo nanocubes is about 10.6 nm, suggesting good monodisperse property in FBS (Figure 2C). The composition analysis of PtPdMo was performed using X-ray photoelectron spectroscopy (XPS) in Figures 2D–F, which indicated Pt 4f, Pd 3d, and Mo 3d valance state, suggesting successful doping to form ternary alloy. Surface defect is an efficient method to increase the active site of a catalyst (Chang et al., 2010; Fujita et al., 2012), which could be produced by lattice distortion or doping, such as engineering high density of oxygen vacancy defects on CeO_2_ nanocubes, or doping CeO_2_ with Cu^2+^ to produce Cu_x_Ce_1−x_O_2_ nanospheres (Lawrence et al., 2011; Yang et al., 2014). Thus, in this work, the PtPdMo nanocubes with dislocation structure can enhance catalytic antioxidant abilities by the engineered surface defects and increased active sites.

### Surface Defects Increased the Peroxidase Activity of PtPdMo Nanocubes

**Figure 3:**
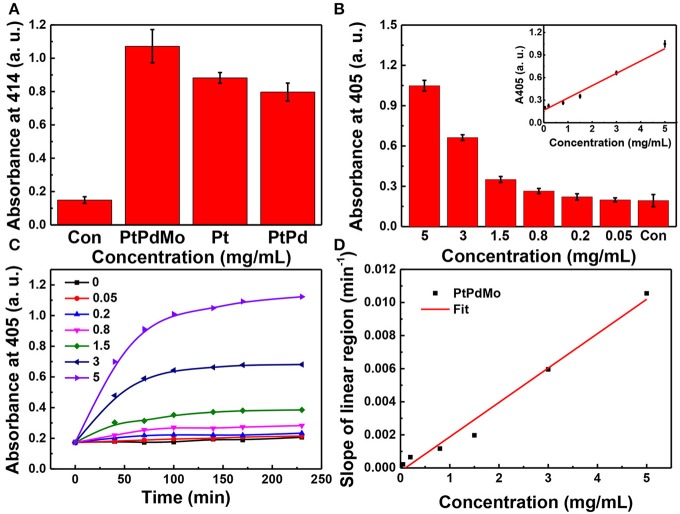
**(A)** Absorption at 414 nm of ABTS working solution after incubated with Pt, PtPd, and PtPdMo solution for 1 h. When the absorbance at 414 or 405 nm of ABTS^+.^ is greater, the sample shows a higher peroxidase-like activity. **(B)** Concentration-dependent absorbance at 405 nm after incubation with PtPdMo for 140 min. **(C)** Time-dependent absorbance profiles at 405 nm for PtPdMo at different concentrations. **(D)** The slope in linear region (catalytic rate) at different concentrations (Data was presented as mean ± SD).

The peroxidase activity of PtPdMo was tested using ABTS as an indicator (Figure 3). ABTS can be converted into ABTS^•+^ with green color by H_2_O_2_ in the presence of peroxidase. The absorbance at 414 nm is in accordance with the extent of reaction. Therefore, Pt, PtPd, and PtPdMo were incubated with the reaction system to investigate the catalytic ability as peroxidase analog. As displayed in Figure 3A, PtPdMo exhibited stronger catalytic ability compared with Pt and PtPd after 1 h incubation. Besides, catalytic ability was in linear relation to the concentration of PtPdMo (Figure 3B). Time-dependent reactions of PtPdMo in different concentrations were also performed in Figure 3C, and the reaction rate in linear region was calculated in Figure 3D. The catalytic reaction rate was concentration-dependent from 0.05 to 5 mg/mL within 70 min. The results are in agreement with previous work that ternary alloy showed superior catalytic property to H_2_O_2_ than Pt, and PtPd (Wang J. Y. et al., 2018).

### PtPdMo Inhibited Cellular ROS Production After Radiation

**Figure 4:**
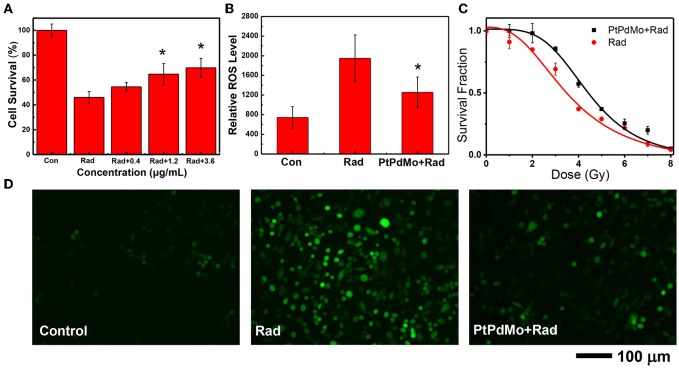
**(A)** The survival of CHO cells after 4Gy radiation, with the treatment of PtPdMo. **(B)** Intracellular ROS level detected by DCFH-DA after 4Gy radiation. **(C)** Radiation dose-dependent colony formation assay. **(D)** Cell fluorescent images after incubated with DCFH-DA at 24 h after 4Gy radiation (Data was presented as mean ± SD, ^*^p < 0.05 compared with radiation group by one-way ANOVA).

The *in vitro* radiation protection was investigated on CHO cells. PtPdMo nanocubes was not toxic to cells until 48 h incubation at concentrations of 100 and 300 μg/mL. Cell survival after 4Gy radiation was increased by 18.5, 40.7, and 51.8%, respectively at the doses of 0.4, 1.2, and 3.6 μg/mL (Figure 4A). Meanwhile, dose-dependent colony forming was also increased in the presence of PtPdMo (Figure 4C) compared with radiation alone group after 2, 3, 4, 5, and 6 Gy radiation. Intracellular ROS level study using DCFH-DA probe revealed that PtPdMo protected cells by effectively inhibiting radiation-induced ROS production (Figures 4B,D). Based on the analysis by flow cytometer, ROS level in PtPdMo treated cells decreased by 35.5% compare to radiation alone group, suggesting high catalytic ability.

Catalytic nanomaterials have high activity of scavenging ROS (Marković et al., 2001; Panikkanvalappil et al., 2013; Zhang et al., 2015b; Xu et al., 2017). It is reported that 35% ROS can be scavenged by Pd nanocubes after H_2_O_2_ stimulation (Ge et al., 2016). Other antioxidant nanomaterials such as CeO_2_ and Ag NPs, also showed significant radioprotective effects *in vitro*. The viability of HacaT cells decreased to 63.0% after UVB radiation, however viable cells increased up to 94.6% with the pretreatment of Ag NPs (Arora et al., 2015). In addition, CeO_2_ nanoparticles protected cells from radiation-induced (20 Gy) damages and showed rarely death rate (1%) at the dose of 10 nM, in strong contrast with radiation alone group (15%)(Colon et al., 2010). Moreover, PEGylated CeO_2_ nanoparticles showed better radioprotective effects than naked CeO_2_, and improved cell viability by 33.3% after 20 Gy radiation on human liver cells (Li et al., 2015). Our previous work showed that PtPd nanocubes decreased the ROS level by 14.8% after 4Gy radiation (Long et al., 2019). Besides, PtPdRh nanocubes can reduce radiation-induced ROS by 18.8% (Wang J. Y. et al., 2018). However, the present PtPdMo nanocubes exhibited superior ROS scavenge ability (35.5%) via doping induced surface defect, therefore prevented ROS damages to cells and increased the survival rate after radiation.

### PtPdMo Increased Survival Rate and Alleviated Oxidative Stress *in vivo* After Radiation

**Figure 5:**
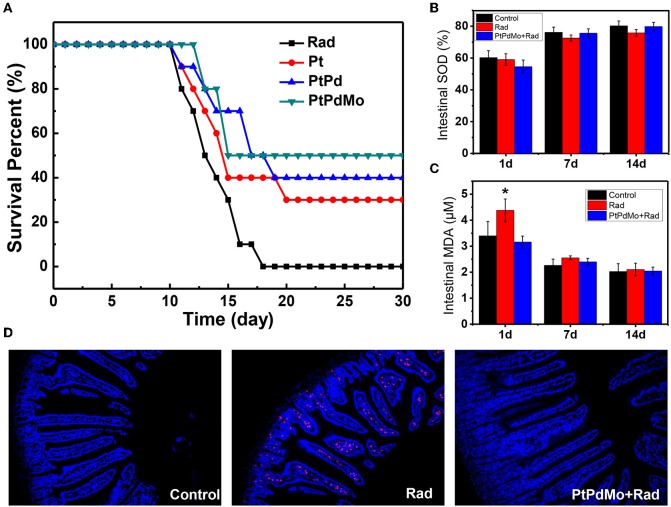
**(A)** Animal survival fraction after 7.2Gy whole body radiation during 30 days. **(B)** Intestine SOD activity at 1 and 7 days after radiation. **(C)** Intestine MDA level after radiation. **(D)** Cell apoptosis in intestinal epithelium using TUNEL assay 7 days post radiation (Data was presented as mean ± SD, ^*^p < 0.05 compared with control group by one-way ANOVA).

The radioprotective effects of PtPdMo were further investigated on C57 male mice. As shown in Figure 5A, after being subjected to 7.2 Gy irradiation, no animals survived 17 days later. However, survival rate increased with nanocubes treatment. Ternary alloy PtPdMo showed highest survival rate (50%) compared with its Pt and PtPd counterparts, which were 30 and 40%, respectively. The radioprotective mechanism was next investigated on radiation-induced gastrointestinal system damages. In Figure 5B, SOD activity diminished 1 day post radiation, but 7 and 14 days later PtPdMo treatment group exhibited better recovery than control group (Figure 5B). Intestine lipid peroxide increased dramatically 1 day after radiation, while PtPdMo treatment declined MDA approaching to the normal level (Figure 5C). MDA decreased 7 and 14 days after radiation, and mice in PtPdMo treated group showed lower MDA level than radiation alone group. Besides, radiation-induced intestinal cell apoptosis was detected by TUNEL assay (Figure 5D). Apoptotic cells spread widely in intestinal epithelium (rad spots) post radiation. However, PtPdMo protected gastrointestinal system by inhibiting cell apoptosis and villi damages.

As known, the survival rate after radiation is a crucial indicator for evaluating the radioprotective effects, which is also influenced by the exposure sites and imposed doses. CeO_2_ nanoparticles increased the survival rate to ~15% after irradiated a total dose of 30 Gy in the thoracic ventral area (Colon et al., 2009). Our previous work revealed that MoS_2_ nanodots as an effective radioprotectant could increase the survival rate up to 79% after 7.2 Gy total body radiation (Zhang et al., 2016b). The radiation protection effects of catalytic material are based on the antioxidant property that could scavenge accumulated ROS to alleviate oxidative stress. The tissue SOD is a counterbalance to MDA level, which is the product of lipid peroxide and can be used as an oxidative stress indicator. In this work, intestine MDA was efficiently inhibited with higher SOD activity at 1 day with the pretreatment of PtPdMo, thereby improving the recovery from radiation damages 7 days later and inhibiting intestinal cell apoptosis. The tendency is agreement with the *in vivo* study of Graphdiyne-BSA NPs, which also showed a surge of MDA in liver and lung after 1 day but recovered to normal at 7 days with Graphdiyne-BSA NPs treatment (Xie et al., 2019). Herein, based on our results PtPdMo nanocubes with high defects or dislocation could be applied as an effective antioxidant adjuvant for radiation protection, and introducing active sites by defect-generating to improve antioxidant properties can also be employed to various catalysts (Kozachuk et al., 2014).

### PtPdMo Protect Intestinal Tissue From Radiation Damages

**Figure 6:**
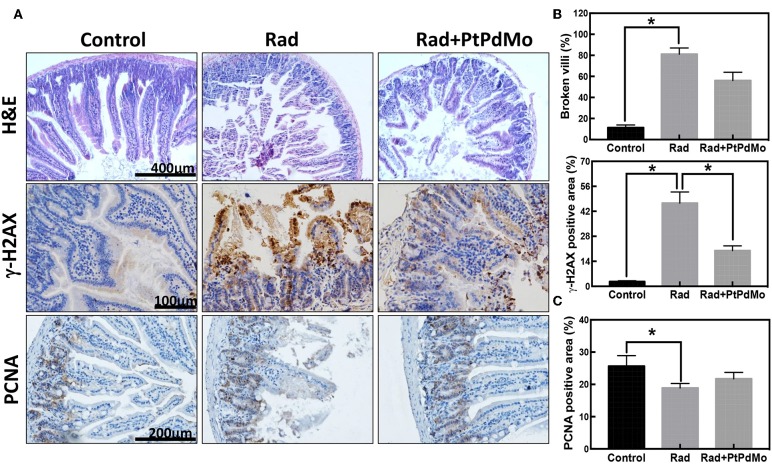
**(A)** Pathology study carried 7 days after radiation using H&E staining. **(B)** Immunochemistry staining of γ-H2AX and **(C)** Immunochemistry study of PCNA 7 days after radiation. Positive area was calculated using IHC tool box in ImageJ software (Data was analyzed using one-way ANOVA, and presented as mean ± SD, ^*^p < 0.05). The mice were exposed to ^137^Cs-γ ray with dosage of 7.2 Gy, and the dose rate was 1.0 Gy/min.

To further investigate the protection mechanism of PtPdMo, intestinal damages were identified by immunochemistry. Pathology study was performed by H&E staining as displayed in Figure 6A. The radiation alone group showed loss of intestinal villi with fragmentary structure, however intestinal villi exhibited better integrity after PtPdMo treatment. Ionizing radiation is known to result in DNA double stranded breaks (DSBs), characterized by phosphorylation of γ-H2AX protein. Therefore, we employed anti-γ-H2AX antibody to detect radiation-induced DSBs in intestinal epitheliums (Figure 6B). Results showed that intestinal villi were severely damaged with high γ-H2AX expression after radiation compared with control group. However, positive area of γ-H2AX reduced significantly in PtPdMo group and statistical differences were detected compared to radiation alone group. Proliferating cell nuclear antigen (PCNA) resides in the transiently amplifying (TA) cells that migrate downwards to give birth to Paneth cells, or upwards to villus and differentiate into enterocytes. Ionizing radiation inflicted epithelium cells and diminished the population of TA cells compared with normal intestine (Gong et al., 2016). By contrast, the number of TA cells was increased with PtPdMo treatment, indicating better repairing ability after damages (Figure 6C).

Radiation induced gastrointestinal syndrome is typical side effects due to the intestinal stem Therefore, intestinal damage is a main focus of radiation protection studies. For example, CeO_2_ nanoparticle was reported to protect the gastrointestinal epithelium against radiation-induced damage by scavenging free radicals and increasing the SOD2 expression before radiation insult (Colon et al., 2010). Additionally, C_60_(OH)_24_ nanoparticles significantly ameliorated the radiation induced tunica mucosa damage and congestion of blood vessel in intestine, and showed 60% survival rate after radiation (Vesna et al., 2016).

### PtPdMo Ameliorated Radiation-Induced Hematopoietic and Multi-Organ Damages

**Figure 7:**
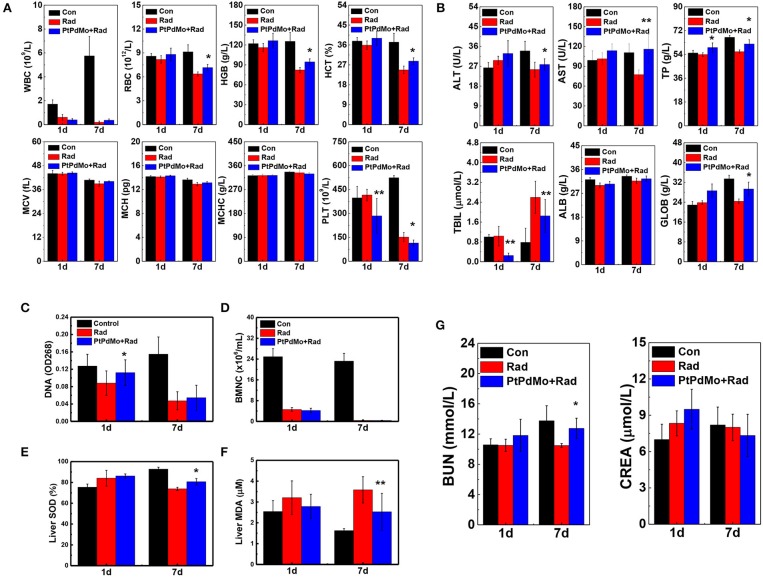
**(A)** Hematology panels, Red blood cells (RBC), white blood cells (WBC), platelets (PLT), mean corpuscular hemoglobin (MCH), mean corpuscular hemoglobin concentration (MCHC), mean corpuscular volume (MCV), hemoglobin (HGB), and hematocrit (HCT) were assessed at 1 and 7 days post radiation. **(B)** Serum biochemistry analysis at the same time points after radiation, parameters include aminotransferase (ALT), aminotransferase (AST), total protein (TP), albumin (ALB), globulin (GOLB), and total bilirubin (TBIL) **(C)** Bone marrow DNA level **(D)** Bone marrow nucleated cells **(E)** liver SOD activity and **(F)** liver MDA level at 1 and 7 days after injury. **(G)** Blood urea nitrogen (BUN) and creatinine (CREA) were tested for renal damage changes of irradiated mice by PtPdMo nanocubes treatment or not (Data was presented as mean ± SD, ^*^p < 0.1, ^**^p < 0.05, compared with radiation group by one-way ANOVA).

Hemopoietic system as another radiosensitive tissue was also investigated at 1 and 7 days after exposure. Peripheral blood was analyzed with the blood cell counter by parameters including white blood cells (WBC), erythrocyte (RBC), hemoglobin (HGB), hematocrit (HCT), mean corpuscular volume (MCV), mean corpuscular hemoglobin (MCH), mean corpuscular hemoglobin concentration (MCHC), and platelet (PLT). Radiation induced apparent blood cell deficits compared with healthy animals especially for WBC, RBC, HGB, HCT, and PLT (Figure 7A). Moreover, based on the peripheral blood analysis, damages to hemopoietic system aggravated after 7 days, which was also supported by the results of bone marrow DNA and nucleated cell tests (Figures 7C,D). However, hematopoietic damages were ameliorated with the treatment of PtPdMo. Multi-organ injuries resulted from ionizing radiation were assessed by serum biochemistry and liver oxidative stress. Serum biochemistry study displayed in Figure 7B focused on panels including aspartate transaminase (AST), alanine transaminase (ALT), albumin (ALB), total bilirubin (TBIL), globulin (GLOB), and total protein (TP). The abnormal level detected in blood serum indicated that ionizing radiation caused multiple-organ injuries, especially to liver. The level of AST, ALT and TBIL changed distinctly for radiation only group, suggesting liver injury induced by ionizing radiation. We also tested urea nitrogen (BUN) and creatinine (CREA). The results, in Figure 7G, showed that ionizing radiation also caused renal injuries. As a contrast, PtPdMo effectively reduced liver damages by alleviating oxidative stress, which was in consist with lipid peroxide results presented in Figure 7F. Meanwhile, SOD activity was also enhanced by PtPdMo treatment compared with radiation only group (Figure 7E).

The hematopoietic system is highly sensitive to radiation assault. Radiation-induced hematopoietic syndrome is characterized by hemopoietic progenitor cell damages and blood cells depletion, however the number of BMNC and peripheral blood cells recovered to a large scale 7 days later with the treatment of PtPdMo nanocubes. All the *in vivo* studies indicate that PtPdMo nanocubes with surface defects can scavenge the overall ROS in body, ameliorate the oxidative stress and therefore increase the survival rate.

### Discussion About the Medical Studies of Nanomaterials

In recent years, radiotherapy combined with nanomedicine to overcome limitations during cancer treatment has been showing great advantages compared with traditional regimes (Song et al., 2017; Wang H. et al., 2018). The engineered nanomaterials can deliver radioisotope precisely to solid tumor, reducing the risk of exposure to normal tissues (Cao et al., 2004; Chunfu et al., 2004; Zhang et al., 2015a; Chao et al., 2016). Besides, materials with high-Z elements such as gold nanoclusters have shown high efficiency as radiosensitizers to suppress tumor malignancy (Zhang et al., 2012, 2014, 2015a,b). Moreover, pH responsive nanomedicine is also developed to undermine the hypoxia environment inside tumors (Fan et al., 2015; Ge et al., 2016; Yi et al., 2016; Zhu et al., 2016). As a contrast, there is still deficiency in investigations for radioprotective nanomedicine. Most of the studies were focused on cerium oxide (CeO_2_) which is the representative antioxidant nanomaterial (Tarnuzzer et al., 2005; Colon et al., 2009, 2010; Briggs et al., 2013; Kumar et al., 2016). Therefore, it is crucial to develop nanomaterials with high catalytic activity as effective radioprotector and ROS scavenger. In our previous works, a series of nanomaterials with high catalytic ability were designed, and the survival fraction was increased at least to 50% (Zhang et al., 2016b, 2017; Wang J. et al., 2018; Wang J. Y. et al., 2018). However, there is still demand for nanomaterials with better biocompatibility, multiple scavenging properties or enzyme-like activities to help treat complex diseases.

Hydrodynamic size is an important indicator for nanomaterials to ensure the low toxicity to body. PtPdMo nanocubes employed in this study has the hydrodynamic diameter larger than the threshold of renal clearance (5.5 nm), but did not show side effects due to the protection of PVP coating. For future applications, it is necessary to develop ultrasmall materials in sub-5 nm hydrodynamic sizes, which is optimal for renal clearances (Zhang et al., 2014, 2016a,b; Liu et al., 2016) and to identify well-defined surface protection for radiation application.

## Conclusion

In summary, we synthesized the PtPdMo nanocubes and increased the antioxidant property by creating surface defects. The effects of radiation protection were explored, especially for intestinal epithelium and hematopoietic system. It was found that PtPdMo nanocubes increased the survival fraction after lethal dose of radiation compared with the Pt, PtPd, and control groups. Furthermore, PtPdMo nanocubes protected intestinal epithelium from apoptosis and oxidative stress. Radiation induced DNA DSBs were significantly inhibited with PtPdMo treatment, which also preserved intestinal TA cells to promote intestine recovery from radiation damages. Besides, bone marrow DNA and BMNC damages were alleviated in the presence of PtPdMo nanocubes. Moreover, the radiation-induced peripheral and liver damages were also recovered with PtPdMo treatment, suggesting great potential as a radioprotector.

## Data Availability Statement

The raw data supporting the conclusions of this article will be made available by the authors, without undue reservation, to any qualified researcher.

## Ethics Statement

The animal study was reviewed and approved by the Animal Studies Committee of the Institute of Radiation Medicine.

## Author Contributions

WL and X-DZ: conceptualization. J-YW and Y-MS: data curation. XM, JW, JC, and Y-MS: formal analysis. WL: funding acquisition. WL and FX: investigation. XM, FX, and SS: methodology. X-DZ: project administration. SS: software. JY and HW: supervision. J-YW and JW: validation. WL: writing—original draft. J-YW, JY, and HW: writing—review and editing.

### Conflict of Interest

The authors declare that the research was conducted in the absence of any commercial or financial relationships that could be construed as a potential conflict of interest.
